# Mutual Shaping in Swarm Robotics: User Studies in Fire and Rescue, Storage Organization, and Bridge Inspection

**DOI:** 10.3389/frobt.2020.00053

**Published:** 2020-04-21

**Authors:** Daniel Carrillo-Zapata, Emma Milner, Julian Hird, Georgios Tzoumas, Paul J. Vardanega, Mahesh Sooriyabandara, Manuel Giuliani, Alan F. T. Winfield, Sabine Hauert

**Affiliations:** ^1^Bristol Robotics Laboratory, Bristol, United Kingdom; ^2^University of Bristol, Bristol, United Kingdom; ^3^University of the West of England, Bristol, United Kingdom; ^4^Toshiba Research Europe Limited, Bristol, United Kingdom

**Keywords:** users, mutual shaping, swarm robotics, firefighting, rescuing, storage organization, bridge inspection, responsible research and innovation

## Abstract

Many real-world applications have been suggested in the swarm robotics literature. However, there is a general lack of understanding of what needs to be done for robot swarms to be useful and trusted by users in reality. This paper aims to investigate user perception of robot swarms in the workplace, and inform design principles for the deployment of future swarms in real-world applications. Three qualitative studies with a total of 37 participants were done across three sectors: fire and rescue, storage organization, and bridge inspection. Each study examined the users' perceptions using focus groups and interviews. In this paper, we describe our findings regarding: the current processes and tools used in these professions and their main challenges; attitudes toward robot swarms assisting them; and the requirements that would encourage them to use robot swarms. We found that there was a generally positive reaction to robot swarms for information gathering and automation of simple processes. Furthermore, a human in the loop is preferred when it comes to decision making. Recommendations to increase trust and acceptance are related to transparency, accountability, safety, reliability, ease of maintenance, and ease of use. Finally, we found that mutual shaping, a methodology to create a bidirectional relationship between users and technology developers to incorporate societal choices in all stages of research and development, is a valid approach to increase knowledge and acceptance of swarm robotics. This paper contributes to the creation of such a culture of mutual shaping between researchers and users, toward increasing the chances of a successful deployment of robot swarms in the physical realm.

## 1. Introduction

Swarm robotics uses a large number of robots that follow simple rules and use only local interactions to achieve seemingly complex group behaviors (Şahin, 2004; Brambilla et al., 2013). It has been demonstrated as a useful technology under laboratory conditions (Bayindir, 2016). Swarms have a wide array of application areas such as search and rescue (Penders et al., 2011), construction (Werfel et al., 2014), and space exploration (Vassev et al., 2012). Despite a lengthy list of real-world applications, there is a lack of research into the practicalities of swarm robot deployment (Bayindir, 2016). One important factor that has not yet been properly investigated is public perception and likelihood of acceptance of robotic swarm products by users. The media and entertainment industry have depicted swarm robotics as something to be feared, according to Hamann (2018). This is troubling for the field since 21% of the respondents to a report by Ipsos MORI and the Royal Society (2017) said that their perception of AI was heavily influenced by mainstream media and entertainment (including science fiction). Additionally, a survey by the European Commission in 2017 (Eurobarometer, 2017) found 37% of respondents felt uncomfortable with robots assisting them at work. In relation to swarm robotics, it is not known what workers expect from robot swarms, and whether they would be comfortable working alongside them.

This work aims to address this gap by engaging with potential users of future swarm robotics systems. We create a two-way relationship between researchers and users which will encourage and inform mutual shaping of the technology. In particular, users acquire knowledge about the technology from researchers, and researchers learn about potential exploitation of the technology from users, hence critically revising the technology. In this paper, we present qualitative results from user participatory design style discussions with a total of 37 participants across three different sectors: fire and rescue, storage organization, and bridge inspection. Our goal during the three studies was to identify the challenges users face in their profession, learn from their reactions to possible assistive swarm systems, and discover any barriers to the system's acceptance, as well as to introduce them to the field of swarm robotics. By incorporating users in the early stages of research and development of swarm robotics systems, we aim to increase their adoption of the technology. This is essential to successfully implement such systems in real-world applications that have economic and societal benefits (Winfield and Jirotka, 2018).

## 2. Related Work

There has been an abundance of research in human-robot interaction research in industrial settings (Berg et al., 2019) and in search and rescue (Murphy, 2004). There has also been important work into understanding what users need from search and rescue technologies such as Adams (2005), Driewer et al. (2005), Yanco et al. (2006), or Harbers et al. (2017). User studies help shape the technology itself and inform the requirements that the design processes should follow to produce a successful robotic product for an application. Successful here would mean working well alongside the human workers. For this, roboticists should investigate the attitudes of these workers toward robotics. Authors of studies such as Katz and Halpern (2014) have conducted interviews with people (in this case, students) about their opinions on the suitability of robots for various occupations. For example, it was found that the appearance of the robot played a part in the human worker's attitudes toward it and their perceptions of its likely performance. Similarly, investigations have been conducted into the perceptions of robot capability and how desirable they are to workers (Takayama et al., 2008).

There is a lack of similar research into swarm robotics. There has been some research into human-swarm interaction (Couture-Beil et al., 2010; Nagi et al., 2012, 2014; Pourmehr et al., 2013; Kolling et al., 2016; Nam et al., 2019; St-Onge et al., 2019). However, the attitudes, perceptions and desires of workers for swarms has not yet been researched (to our knowledge). Existing research into how humans feel about swarms has focused on the psychophysiological response rather than opinions or expectations. For example, Podevijn et al. (2016) studied the effect increasing the size of robot groups had on the stress and anxiety of participants and found that a higher number of robots provoked a heightened response.

While a wealth of literature exists mentioning the sectors in this project, few describe a swarm system that operates in reality. For example, a range of robots have been developed for fire and rescue (see Murphy, 2014; Delmerico et al., 2019). Of these, the most complete swarm system is the GUARDIANS project (Penders et al., 2011). The GUARDIANS project developed a swarm of autonomous robots to assist firefighters with navigational support in low vision scenarios. In the context of the second study, storage organization, robots have been used in warehouses successfully for a number of years (Bahrin et al., 2016). Swarm algorithms for typical tasks in a storage facilities have also been developed such as cooperation when lifting objects (Wilson et al., 2014). The final study, bridge inspection, robotic solutions have generally used single UAVs (Murphy et al., 2011; Khaloo et al., 2018) and computer vision to process captured images (Yeum and Dyke, 2015). Swarm based mapping algorithms such as Kegeleirs et al. (2019) have been proposed which could be used for this application.

This work extends the current state of the art by examining the attitudes of users to real-world deployments of robot swarms. Based on this, we propose design principles that can facilitate the development of swarms for real-world applications, by increasing user acceptance of swarm robotics technology.

## 3. Methodology

User studies were designed following the principles of mutual shaping, a framework which aims to create a bidirectional relationship between users and technology developers to incorporate societal choices in all stages of research and development. This approach facilitates the creation of “more socially robust, responsive, and responsible robots” (Šabanović, 2010). In particular, the mutual shaping structure successfully applied by Winkle et al. (2019) was used to structure our three studies. Winkle et al. propose to split up mutual shaping sessions in three main parts:

1. **Pre-demonstration Discussion** to understand participants' initial ideas on the topic before being given information,
2. **Project Presentation and Robot Demonstrations** to introduce participants to the topic of the session by giving an overview of the state of the art, aims of the project, an explanation of the topic, and (perhaps) a robot demonstration, and
3. **Post-demonstration Discussion** for participants to give their opinions to researchers about the topic as well as their requirements to advance in the development of the particular technology in discussion.

We adapted this methodology to the topic of swarm robotics. A summary of the resulting common structure that we followed across the three studies is given below. For a complete description for each structure, please see the Supplementary Materials.

**Figure 1:**
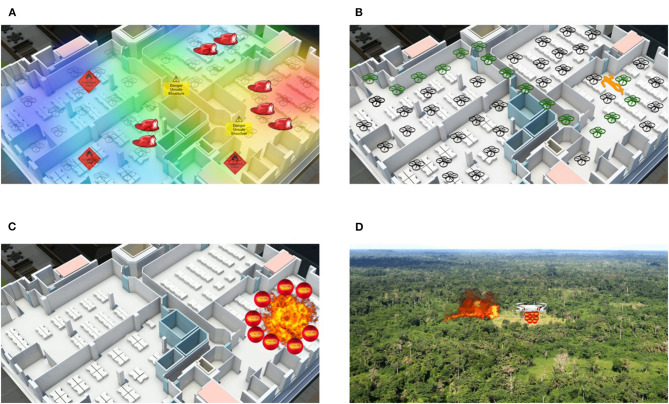
Possible application scenarios shown in the study with fire and rescue services. **(A)** The swarm collects information in a building on fire. **(B)** The swarm shows exit routes to persons in the building or creates communication links inside a building on fire. **(C)** The swarm extinguishes a fire in a building. **(D)** The swarm extinguishes a wildfire in a forest. Indoor map image modified from Valzania and WRLD3D (2019). Forest image belongs to public domain (CC0).

**Figure 2:**
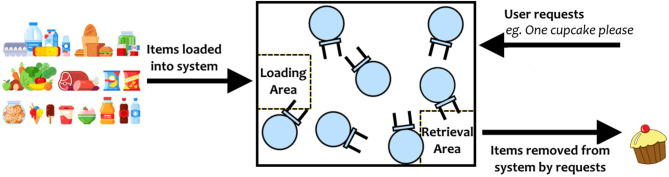
The swarm system described to the interviewee. The storage organization system is described as automatically sorting stock input, and producing items upon user request. How the swarm operates within the box is not described.

**Figure 3:**
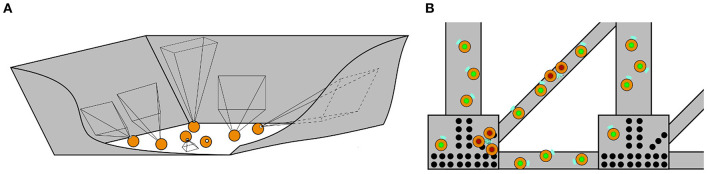
The two scenarios discussed in the bridge inspection sessions, robots are shown in orange while the bridge structure is shown in gray **(A)**—A swarm of robots which captures many images of enclosed spaces to produce 3D models **(B)**—A swarm which traverses the exterior of the structure performing damage detection. In this illustration individual robots indicate detected damage by changing visual indicators from green to red.

1. **Art of their profession:** Participants are asked about their area of work, typical tasks/procedures in their jobs, tools/equipment they normally use, challenges they face, and their attitudes toward robots in their workplace. This first part allowed researchers to understand the art of the participants' profession, as well as their initial attitudes toward the use of robots at work.
2. **Introduction to swarm robotics and possible scenarios of application:** An explanation of swarm robotics is given for participants to learn about this technology and the current state of the art of robot swarms in their fields. Then, a series of imaginary-but-possible scenarios related to their fields, where robot swarms could be applied, are described by the researchers.
  - **Fire and rescue study**: In this study, four different scenarios are presented to participants (see Figure 1). These scenarios show swarms collecting information in a building on fire, creating communication links or finding exit routes in the building, extinguishing the fire in the building, and extinguishing a wild fire in a forest.
  - **Storage organization study**: An out-of-the-box swarming system is described to participants (see Figure 2). This system can sort stock efficiently, provide information about the stock to the user, and retrieve items.
  - **Bridge inspection study**: In this study, two scenarios are given to participants (see Figure 3). These scenarios describe swarms being released in a bridge and creating a 3D model of it by taking individual pictures, and swarms exploring the bridge to detect damage.
3. **Discussion of scenarios:** Finally, a group discussion of the previous scenarios, and others suggested by participants, is held between participants and researchers. In the discussions, the topics of acceptance, levels of autonomy, trust, swarm robotics vs. single robot approach, opportunities for swarm robotics in their fields and their concerns are brought up by the researchers. This part was used to identify the way forward to successfully apply robot swarms to their fields in the future.

For the study with fire and rescue services, focus-group-style sessions were chosen to have teams with different roles discussing the topics and contrasting opinions during the same session, as opposed to interviewing firefighters individually. A total of 23 participants from three different fire and rescue services were recruited, with experience ranging from 1 to 20 years of service, as they verbally stated. Participant recruitment was done via email, word of mouth and on-site visits to fire and rescue services in the UK and Spain by the researcher in charge of this study. Participants were given an information sheet with a full description of the study and the focus group. They were also asked to sign a consent form to participate in the study and accept audio recording of the session, complying with university ethics regulations for experiments with human participants. Ethical approval was given by the University of the West of England. Three focus groups were held, one per service. The first focus group consisted of six participants from a UK fire and rescue service. There were participants working in the risk intelligence unit, IT, group management, media communication, operational effectiveness in instant ground, technology management, and drone piloting. This focus group was held at the Bristol Robotics Laboratory. In the second focus group, four firefighters from a fire station belonging to another UK fire and rescue service came to participate. This focus group was also held at the Bristol Robotics Laboratory. Finally, a third focus group was organized at a Spanish fire station with the participation of 13 firefighters. The diversity in participants allowed for the opinion of firefighters with real firefighting and rescuing experience as well as people working in more technical fields related to development of processes. A pre-questionnaire and post-questionnaire was handed out at the beginning of the session (before any discussion could occur), and at the end of the session, respectively. Both questionnaires had the same questions, which are listed in Supplementary Materials. These questionnaires were used to measure the impact that the mutual shaping sessions had on participants.

In the storage organization study, an interview-style session was used rather than focus groups. This method was chosen because the interviews took place in the workplace to make arrangements easier for the subjects and to include an inspection of the storage space. The variation in locations and working hours meant that collecting participants together in a focus group was not possible. Interviewees were found mostly via email but also by word of mouth. A total of 25 introduction emails were sent out to 25 possible interviewees who fit the use case briefs. The following use case categories and sub-categories were contacted:

1. **Retail:** Charity shops; Shoe shops; Book shops; Jewelery shops.
2. **Food:** Supermarket; Food banks; Cafés.
3. **Industrial warehouses:** Supermarket depot; Retail e-businesses; Aerospace factories.
4. **Supplies in remote locations:** Space missions; Scientific (e.g., Antarctic) treks and laboratories.
5. **Other:** Museums; Independent shops; Stationary shops.

A total of eight interviews were conducted from six distinct use cases: a supermarket; a charity shop; a charitable food bank; a museum with a café and a gift shop; a large-scale industrial warehouse; and the space industry (specifically manned missions to other planets or space stations). Ethical approval was received from the University of Bristol on the condition of consent from interviewees, no audio recordings, and anonymous information gathering. For this reason, it was emphasized in the email that no recordings would be taken during the interviews, only handwritten notes, for which the interviewees gave permission for their answers to be used on consent forms. Attached to the introduction email was an information sheet that was written just for this purpose, explaining what swarm robotics is and what the benefits are of swarming systems. The interviews were performed on a semi-structured basis with a framework of key questions but the flexibility to move around topics that the interviewees wanted to discuss. All of the questions were asked without visual aids and spoken either in person or over the phone.

The bridge inspection study was conducted using focus groups because all participants were in the same industry, and it allowed the data to be collected more efficiently than with individual interviews. Ethical approval was given by University of Bristol. This required that only hand written notes were used to record participants' responses and that all participants remained anonymous. Four companies within the UK bridge inspection industry were contacted via email directly. Two different companies responded to the request leading to two sessions with six participants in total. All participants were engineers and inspectors involved in the management of bridge structures or the inspection process itself. The focus groups were executed in a semi-structured fashion. One researcher lead the discussion while another made handwritten notes. Once participants had read an information sheet, and were happy to participate, a consent form was signed and the session could begin.

This paper aims to be a first step toward understanding requirements of robot swarms through a mutual shaping methodology, built on in-depth, qualitative analysis of interviews with users to identify common themes across the three studies. It does not intend to be a quantitative analysis of user needs, which would require a different methodology based on broader sampling and recorded demographic data. In this sense, questionnaires were not used in the storage organization and bridge inspection studies because they had fewer numbers of participants, and were shorter in duration, due to the nature of the professions targeted.

## 4. Results

### 4.1. Fire and Rescue Study

Below we combine the results from the three focus groups held with fire and rescue services to summarize their current processes, challenges, and attitudes toward using robots in fire and rescue.

#### 4.1.1. The Art of the Profession

Nowadays, firefighters are in charge of many different tasks, not only firefighting. Apart from fires, they go to vehicle collisions, major transport incidents, and hazmat incidents. They also do urban search and rescue (when a building collapses), mine rescue, water rescue, animal rescue, and community-based roles to educate the public. When facing incidents, the first things they do are related to gathering as much information as possible for their risk assessment decision-making processes. Before handling the incident, firefighters perform quick checks to guarantee their safety first, e.g., they assess that the structure is safe to operate, or locate access/exit points. After enough information has been collected, firefighters start actions, i.e., firefighting or rescuing, until the incident is completely handled. Then, a fire investigation to discover the cause of the incident might take place. When participants were asked during the focus groups about the current tools they use for firefighting and rescuing, they stated that all tools they use are not automated, but require human operation. A summary of the tools that they currently use is given below:

- **Sensors and actuators fitted to buildings**. They said that smoke detectors, heat detectors, and water sprinklers assist them before/during firefighting.
- **Thermal imaging cameras**. They are used to create a map of temperatures to look for the source of the fire and casualties. They are particularly useful to predict what is behind areas with difficult access in buildings. Firefighters highlighted how this type of camera improves their performance:
  “Thermal image cameras are one of the great tools we've got. So we can actually see in darkness and make our way around.”
- **Hydraulics**. They use hydraulic tools to cut through things.
- **Maps in the fire truck**. These maps are used to locate possible risks, water supplies, or weather conditions before arriving to the incident.
- **Radio-frequency identification (RFID)**. Used for tracking of firefighters.
- **Colcut**^TM^ **cobra**. A system that uses high-pressure water to pierce through walls and fog when they cannot access the room next door.
- **Teleoperated ground robots (QinetiQ^TM^)**. Sometimes they use them to gather information in hazmat incidents:
  “It's got several cameras and a small water jet for testing temperatures rather than actually extinguishing anything. We used to use them with some level of success.”
- **Drones**. Pilots mostly use them to gather information about incidents to make an assessment of scenarios. They have also used them to track people who have gone missing.
- **Air fans**. Used for tactical ventilation, which means creating positive pressure in a building to push smoke out.

#### 4.1.2. Their Challenges

Participants highlighted their main challenges are related to obtaining enough, accurate, and quick information about the incident so that they can feed it into their decision-making processes. In fact, they mentioned they are quite quick in dealing with fires. The challenge for them is to find the location of those fires, and casualties to rescue. They said this is a challenge because many times the information they get is not accurate:

“In a lot of cases even the information you get […] is not always 100% accurate. The address could be wrong or the actual type of fire could be wrong. It will come in as a hedge on fire and you get there to find a fire in a building. Your site has no persons trapped, there's no persons involved in anything at that point, and you get there and you find that there are. There's always a variable. You have minimal information.”

#### 4.1.3. Opinions on Usefulness of Robots for Fire and Rescue

**Figure 4:**
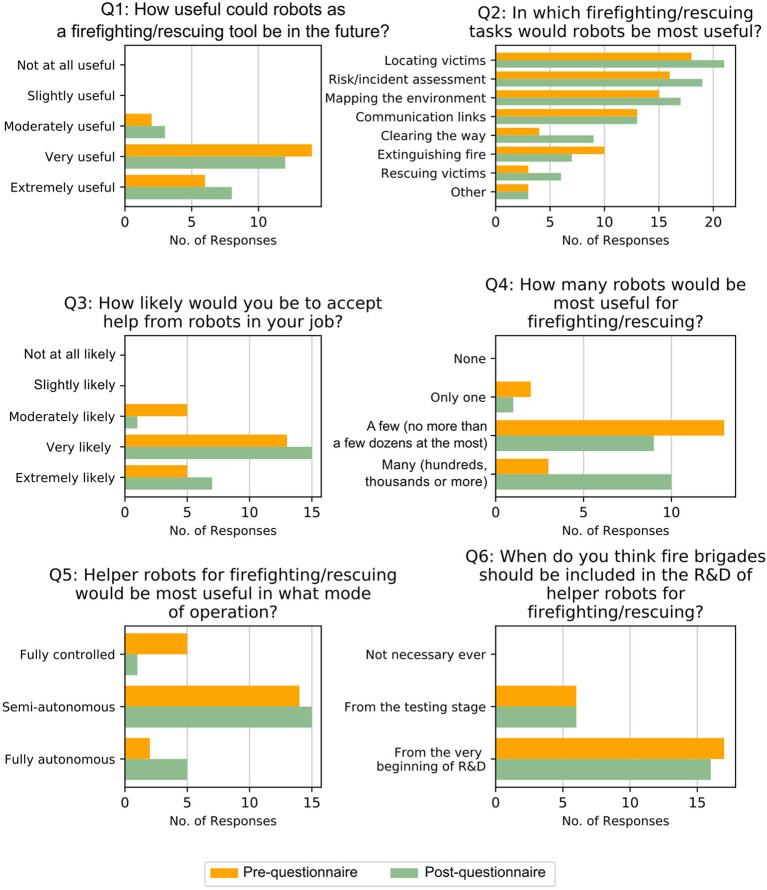
Bar charts of answers to the pre-questionnaire (orange, top bar) and post-questionnaire (green, bottom bar) from firefighters.

Participants could see value in using robots for fire and rescue, as shown in the results of question 1 (“In your opinion, how useful could robots as a firefighting/rescuing tool be in the future?”) in Figure 4. In fact, 20 out of 23 participants ticked *very useful* or *extremely useful* in the post-questionnaire. There was a slight shift from *very useful* to *extremely useful* from the pre-questionnaire to the post-questionnaire, meaning that participants' attitudes were already positive before the sessions. However, they did not think robots should be used for all tasks. Results from question 2 (“In which firefighting/rescuing tasks would robots be most useful?”) in Figure 4 show that information-gathering tasks (locating victims, risk/incident assessment, mapping the environment, communication links) were the ones that participants preferred—they were ticked by over half of the participants. Action-based tasks (clearing the way, extinguishing fire, rescuing victims) were ticked less often, by much less than half of the participants. It is worth highlighting that all tasks but *extinguishing fire* were ticked by the same or more participants in the post-questionnaire, compared to the pre-questionnaire. Hence, participants could see more value in using robot swarms after the session, but thought that extinguishing a fire was too complex to be done by robots. Their preference for information-gathering tasks was also highlighted during the focus groups. Participants said that they would prefer robots doing simple tasks, such as going inside a house, mapping it and coming back to them with information; locating casualties; or sending them to gather information before they get to the incident or searching large areas (e.g., ships).

Participants also highlighted the benefit of using robots to create communication links among firefighters (to coordinate their operations) and between firefighters and casualties (to send them reassuring messages). Indeed, one participant mentioned that their research team was looking specifically at what technology they could deploy into a building to have communication across the whole building. Also, they said that there is poor radio communication in many areas where they go, and they would benefit from deploying relays to establish communications in those areas. Apart from the tasks listed in question 2 of the questionnaire, participants had the choice to specify other tasks that they thought robots could do. In the questionnaires, some participants wrote down the following tasks: Hazardous environment identification, post fire investigation (imagery), bring emergency kit (water, oxygen, food, etc.), protection of victims, rescuer, and habitable zones. During the discussion, even more examples of tasks were raised, such as:

- **Real-time information**. They said it would be useful to have a swarm of robots deployed across the area of the incident to send constant updates.
- **Dangerous or repetitive tasks**. They mentioned they would rather have robots where a human being would be in danger, e.g., hazmat environments. Also, some firefighters mentioned they would like to have robots for repetitive tasks, especially to prevent injuries in firefighters.
- **Finding exit routes**. Participants highlighted their difficulties when dealing with heat stress, because they sometimes get confused/lost inside fires. For that, they thought that having robots finding/lighting up the exit route for them would be particularly beneficial:
  “A building could be like a maze that we're not familiar with […] You want something that could light up […] the floor glow […] something that could glow in the dark.”
- **Tactical ventilation**. Participants gave the example of a swarm of drones using their propellers to perform tactical ventilation to push smoke out of the building.
- **Accessing inaccessible places for firefighters**. Participants said that robots attacking fire in high buildings, where their ladders cannot reach, could be a positive application. They also pictured robots rescuing people from cliffs or water, which sometimes are inaccessible to them.

#### 4.1.4. Opinions on Acceptance of Robots for Fire and Rescue

Participants answered positively to question 3 of the questionnaire (“How likely would you be to accept help from robots in your job?”). All participants but one ticked *very likely* or *extremely likely* in the post-questionnaire, and there was no answer below *moderately likely*, as can be seen in Figure 4. As in question 1, there was also a slight shift toward more positive answers with respect to the pre-questionnaire, but participants were very positive before the session. In fact, during the focus group, participants pointed out that they do not fear robots becoming a replacement for firefighters. Instead, they see them as a tool that could assist them and enhance/complement their operations:

“None of us are negative. We all would like it to happen. Yeah it's just better to have an extra pair of eyes and another person. You just add it to what you're doing visually anyway. Bring it all together, I can certainly see it being really useful for giving us more information.”

When thinking about acceptance from citizens being rescued by robots (or with the help of robots), participants felt that citizens should be educated. They should know what to expect if robots are used for firefighting and rescuing in the future.

#### 4.1.5. Opinions on Robots Swarms for Fire and Rescue

After the session, participants could see how using a large swarm of robots may be the most advantageous option. In question 4 (“In your opinion, how many robots would be most useful for firefighting/rescuing?”), using *many* robots came out as the preferred choice by 10 participants, over *a few* (ticked by nine participants) and *only one* (ticked by only one participant) in the post-questionnaire. It is worth mentioning that two participants did not answer this question, and another one ticked both *a few* and *many*, which was not allowed. Thus, it was not included in the graph of Figure 4. Remarkably, using *many* robots was ticked by only three participants in the pre-questionnaire. Therefore, participants did see the advantages of using a swarm of robots after the sessions.

During the group discussions, participants understood the base principles of swarm robotics, and highlighted their benefits for fire and rescue. In particular, they said that redundancy is one of the key benefits. Most participants preferred to use a robot swarm even if robots could become obstacles (but left this as a requirement for the future). Also, most participants commented that having a large number of robots would be very useful to quickly search an area and gather as much information in the least amount of time as possible:

“The whole idea around swarm is you got some redundancy built in. […] And some of the things we talked about is about location of casualties when it's dark. So deploying small agile devices that can search the rooms at the same time so that firefighters go in and then at least it's a beeping sound, ‘yes, okay, let's prioritize that room.’ […] I think those sorts of things would be our friends.”

#### 4.1.6. Opinions on Autonomy

Their preferred mode of operation for robots is semi-autonomy (15 responses in the post-questionnaire), as seen in results for question 5 (“Helper robots for firefighting/rescuing would be most useful in what mode of operation?”) in Figure 4. In fact, this was the participants preferred mode of operation before they participated in the session, as seen from the results of the pre-questionnaire. The session made them mostly abandon the idea of having *fully controlled* robots. It is worth mentioning that answers from two participants who ticked *fully autonomous* and *semi-autonomous*, and *semi-autonomous* and *fully controlled* were not taken into account. The directive stated multiple answers were not allowed, hence these answers were discarded.

From the group discussion, we understood that participants did not like the idea of robots taking autonomous decisions. They would trust robots carrying out information-gathering tasks or simple actions rather than stepping in the firefighters' decision-making process. Basically, participants feared that the robot system could cause more harm than benefits (e.g., knock-on effects) because there are many variables during fire and rescue, and lives at risk. They gave the example of robots opening up a window and changing the dynamics of the fire due to a change in air flow and the addition of oxygen to it.

In their opinion, robots could support their decision-making processes, but should not be in charge of them. From their comments, they would rather have a human in the loop being responsible for the actions taken when handling the incident:

“If it is autonomous just for firefighting, then I don't think that this is a corporate risk we would accept in this site. You can just imagine the headlines, it can help you and save you a thousand times. But one time it doesn't work properly and we lost a building through fire. Or loss of life even worse. Imagine the headlines: ‘Firefighters sit outside and do nothing while robots sacrifice and get it wrong’. That's a risk that, until the idea is developed and understood more widely, probably we would not accept.”

#### 4.1.7. Opinions on Involvement in the Research and Development Process

The final question was related to when fire and rescue services should be included in the research and development process (“When do you think fire brigades should be included in the research and development process of helper robots for firefighting/rescuing?”). A total of 16 participants answered *from the very beginning*, whereas only six participants ticked *from the testing stage* in the post-questionnaire. One participant did not answer this question, so it does not appear in question 6 in Figure 4. This aspect was not discussed during the focus groups. As seen in the answers to this question in the pre-questionnaire, mostly the same number of participants already thought that fire brigades should be included from the beginning. Their participation in the sessions did not change this opinion.

#### 4.1.8. Requirements for Trust in Robots That Assist in Fire and Rescue

This final section summarizes all the key requirements that participants felt robots used in firefighting and rescuing should have for them to trust these systems:

- **Robots should be easy and quick to learn, deploy, and maintain**. Participants said that setup time should be kept to a minimum to proceed as soon as possible, as well as the number of checks needed to maintain the robots because they do not have enough time. Cost of training should also be low, according to them.
- **Swarms should not become a physical obstacle for firefighters/casualties**. Firefighters described that fires are usually chaotic, with unpredictable conditions.
- **Info given from robots should be relevant and not complex**. Due to the amount of information they manage when dealing with an incident, firefighters said that robots should not give all, raw information, but instead should provide information that is as clear, relevant, and digested as possible.
- **Robot swarms should be reliable**. They stressed the importance of guaranteeing that robots work when deployed, and that the information they provide is accurate. This is because they would make decisions based on what robots tell them:
  “The reliability needs to be on there because again, the first time it fails that's it, you've lost the cause in there. […] Get through those cultural barriers and then you'll find that the actual application implementation of that would be a lot easier.”
- **Robot swarms should be accountable**. Participants said that the data gathered from robots must be stored and timestamped. This is important for their internal investigations.
- **Robot swarms should be safe**. Finally, participants said that robots should guarantee firefighters' safety.

### 4.2. Storage Organization Study

Results for storage organization study were also gathered, using similar quasi-structured questions. One-to-one interviews were used here, rather than focus groups. The answers to the questions and discussions in interviews are given in this section:

#### 4.2.1. Summary of Use Cases

The following are descriptions of the use case stock rooms, based on the answers given by the interviewees when asked how they characterize their day-to-day work:

- **Supermarket** Stock is transported from the depot to the shop where it is moved from the van to the stockroom by employees. The stock is transferred within a large cage on wheels and is kept in this container while in the shop stockroom or stacked on the shelves by employees. There is no stockroom organization system.
- **Charity shop** Donations come in at sporadic, unpredictable times and range across a wide variety of items and quality. Staff initially sort items into two categories: bric-a-brac and clothing and then into further subcategories. The donations are stored in large piles in one corner of the sorting room before they are sorted. The sorting process includes putting clothing on hangers and individually pricing each piece.
- **Food bank** Food donations come in a random order and amount. Volunteers sort the items into categories and write the best-before date on the packaging so it is easily visible. The food is sorted and stored into crates of the same food type and best-before date. When people come in for food donations they make a request for a list of products and the volunteers create a bespoke food package for each customer from the stock in the warehouse.
- **Industrial warehouse** When new stock arrives at the warehouse, before it enters the automated part of the system (which uses a combination of humans and robots to pick and pack items) it is moved using automated guided vehicles (AGVs) into the warehouse storage.
- **Museum** There are multiple parts of the museum building: café; gift shop; event locations; main collection; and archives. All of these areas have their own stock to organize and store. They coordinate their timetables for stock incoming and outgoing using a shared calendar system but do not discuss details beyond this. Extra storage (such as fridges) is hired in for large café events. Volunteers sort through the collection archives, which have over 1 million pieces, and record details about each piece on pen and paper to categorize what is in each storage box.
- **Space missions** On the International Space Station (ISS), astronauts keep track of the food and supplies stock and search the store when they want something. It is predicted that when humans go for extended, residential trips to the Moon or Mars they will need to do this as well. Orders for new supplies need to be made many months in advance because it is difficult to send to them. This means that keeping a careful log of what stock they use and when is important to avoid running out of supplies too early.
- **Large-scale Retailer** The stock is stored in large warehouses from which online orders are packed and shipped. A centralized, robotic system is used to move stock to a conveyor belt where it is transported to human pickers who pack the products.

#### 4.2.2. Current Processes

The following are descriptions of the current, storage organization tasks used by the different use cases, as discussed in interviews. Common processes are grouped together:

##### 4.2.2.1. Inventory

The robots in the system used by the **Large-scale Retailer** automatically scan all stock items and all items are kept in cardboard boxes. This means that there is a constantly updated inventory and corresponding location list. In the **Supermarket**, when a delivery comes in from the depot, a list of what is included in the stock is added to a central database on a computer. The individual items are not checked by the shop employees against the list for errors. This can lead to “negative stock” which is stock that is counted as being in the inventory but never actually arrived. Any items that were on the list of items that arrived but have not been sold or wasted are assumed to have been stolen. In the **Museum**, technology is not used (i.e., no digital record) because there is no network infrastructure in the archives and the volunteers tend to not want to work or train to work with computers. Additionally, it was noted that management was afraid of a risk of losing data due to a computer problem and stated that pen and paper were therefore more reliable. Supplies on **Space** missions such as the International Space Station (ISS) are counted and recorded by crew members. The **Food Bank** and the **Charity Shop** do not keep any inventory or map of their stock and instead they both do stock rotation by eye.

##### 4.2.2.2. Sorting

When donations come into the **Food Bank** or the **Charity Shop**, items are sorted into different categories and stored with other like items. In the case of the charity shop, prices are decided based upon the sorter's personal opinion but reasoning is according to: current trends; brands; quality; judgement. It was stated by one staff member that the reasoning for a price is often just a feeling about how much it's worth.

In the **Supermarket**, similar items arrive together and no re-sorting is done, they are just stored in the stockroom as they are when they arrive. Similarly, in the **Museum**, items are kept in the archive in boxes of mixed types, but no sorting is done. The **Industrial Warehouse** has workers who drive the pallets of new stock from the deliveries into the storage unit of the warehouse where it is collected and sorted by robots into the high-level system. Items in the **Large-scale Retailer** warehouse are sorted into locations depending on speed of movement. Here, fast movement means items are likely to be needed soon such as returned items. Beyond this, items are sorted at random with stock being constantly rearranged by the robots, even overnight, to be more efficiently stored. This is because the items are stored three rows deep so constant rearranging makes it less likely that something will be blocked behind other items for too long to be inefficient.

#### 4.2.3. Challenges With Current Processes

**Figure 5:**
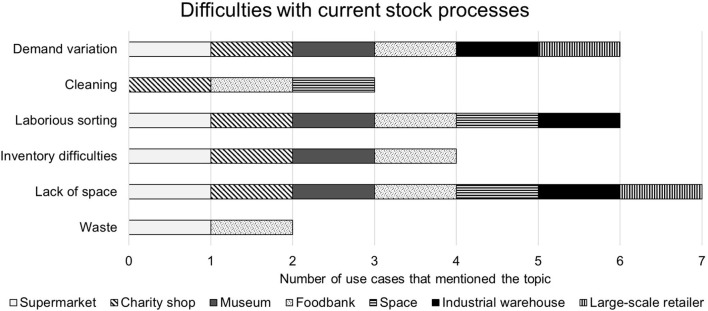
Difficulties encountered in the existing storage organization process, as highlighted by interviewees.

The parts of the current stock organization processes that were highlighted by the interviewees as being negative or difficult are summarized in Figure 5 and given in the following:

##### 4.2.3.1. Sorting issues

All interviewees said that they thought the sorting system that was currently used could be significantly improved and that they wanted to do less sorting themselves. In this way they were all enthusiastic about a technological solution that would mean that they had to do less sorting of stock and/or the process would be quicker and easier. For example, the **Space Industry** experts stated that an astronaut's time was expensive and limited, meaning that sorting stock was considered a waste of resources that should be automated. Similarly, the **Industrial Warehouse** interviewee said that loading speed could be vastly increased to save time and money. This opinion was shared by all of the use cases for similar reasons.

##### 4.2.3.2. Limited space

All of the interviewees said that a disadvantage of their current processes for stock handling was limited space in which to do it. For example, the **Food Bank** storage space was limited, meaning that piles of crates were three rows deep in some places which made it difficult to reach items at the middle or back of the pile. Similarly, in the **Charity Shop**, it was noted by the interviewee that this makes it especially difficult to search the donations for specific items to replenish supplies that are out of stock. The **Space Industry** representatives said that the storage space available is limited because it has to be habitable for humans to manipulate stock. The alternative, which would save money and therefore allow more available storage space, would be to not pressurize or supply oxygen to it, meaning the astronauts would walk around it in their spacesuits. The disadvantage of this is that it takes a long time to get spacesuits on which would also be a waste of time, especially as going and retrieving stored food is likely to be necessary multiple times a day.

The **Industrial Warehouse** employee also stated that there were economic reasons (i.e., cost of land) for keeping the space used for sorting goods to a minimum. The resulting problem is that it is difficult for the AGVs to move around and to prevent traffic jams as goods are being transported from delivery to storage. The **Large-scale Retailer** said that they wanted their system to be more dynamic. This is because the limited space for the robots to move means that when a robot breaks down it can block the way and make some stock areas inaccessible.

##### 4.2.3.3. Demand variation

All interviews except the representatives from the space industry said that demand variation and unpredictable incoming and outgoing stock made it more difficult to do their stock-handling jobs. For example, in the **Supermarket** the stock is more predictable but orders often vary, which can cause the stockroom to become busy and therefore difficult to keep organized. Similarly, in the **Industrial Warehouse** demand can go up and down in the same day, which puts a strain on the current processes due to the need for quickly adapting behaviors.

##### 4.2.3.4. Inventory

The **Museum** stated that mistakes are often made by their volunteers when recording archived items. Similarly, the **Supermarket** said that they do not check stock against the stock list as it comes in so they are not aware of inventory errors but they do occur without their knowledge. No inventory is taken for the **Charity Shop** or the **Food Bank**, which can make the stockrooms hard to search for specific items when they are needed. This is a particular problem for the food bank when a customer requests a specific brand or has an allergy requirement because they do not keep any record of this information. The volunteers have to go to the area of the warehouse with the correct type of food and look at individual items for a matching one. This is laborious and slows down the whole process.

##### 4.2.3.5. Cleaning

The **Food Bank** expressed that they spent a lot of their volunteered time cleaning the products. They resented having to do this and blamed the layout of the warehouse which was difficult to rearrange because of lack of space and heavy crates. The **Charity Shop** also said that cleaning incoming donations was part of their job but that they only did it when an item was likely to get a good enough price to be worth the cleaning time, otherwise they would put it in recycling or scrap materials. They consider cleaning an annoying part of their job, which is why they do not clean most items. The **Space Industry** representatives stated that general cleaning is a necessary part of an astronaut's duties but is considered to be a waste of their valuable time.

##### 4.2.3.6. Waste

The **Supermarket** employee said that due to the way items are stacked together, the items at the bottom of the piles are often damaged. This is particularly common for products where the packaging is irregular in shape which also causes a waste of space due to inefficient packing. The **Food Bank** said that food can go out of date without the staff knowing because they do not have an inventory and cannot see the crates of food that are buried within the pile.

#### 4.2.4. Attitudes Toward the Swarm System

At this point in the interview, the swarm system is described by the interviewer. It is described as a swarm system that automatically sorts stock that is input, and produces items upon user request. The following are the answers given to questions about this swarm system:

##### 4.2.4.1. Features of the storage organization system

**Figure 6:**
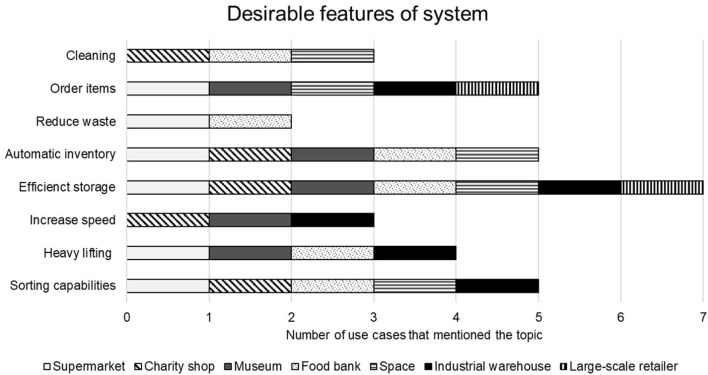
Summary of the stated desired features of the swarm storage organization system in the interviewed use cases.

Many answers were common to more than one user and they are summarized in Figure 6 about the desirable features to be included in a swarm system for storage organization. The most common desirable features were efficient storage (7/7 of use cases), automatic inventory check (5/7 of use cases), and automatic sorting abilities (5/7 of use cases). For example, the **Food Bank** said of the automatic inventory that this would allow them to cater to preferences and allergies more easily. They said that they would like a system that could allow them to do this and cater to other dietary requirements.

The next most useful features of the swarm system stated by 5/7 of use cases was automatically ordering items (e.g., the system would be able to recognize when there was favorable weather conditions or low stock of an item and make orders for new items as a result) and heavy lifting of stock. Finally, the other desirable features of the system stated were: cleaning abilities (3/7), increase loading speed or speed of processes such as inventory or transfer of goods (3/7), reduce wasted products (2/7).

##### 4.2.4.2. Positive comments

**Figure 7:**
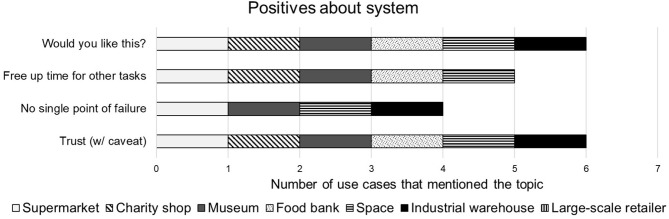
Summary of positive responses to the storage organization system.

The interviewees were given the swarm system and asked for their thoughts about it. The main positive points are given in Figure 7. When specifically asked about how they felt about working alongside swarms of robots in general or compared to working alongside single robot systems the reactions were very positive with 6/7 stating that they would like to have this system in their place of work. Almost all (5/7) of the interviewees expressed positive opinions toward the suggested system for the given reason that it would free up time for some other task. 4/7 interviewees stated that they preferred the swarm system to a similar single robot system because there is no single point of failure in a swarm system.

##### 4.2.4.3. Negative comments

**Figure 8:**
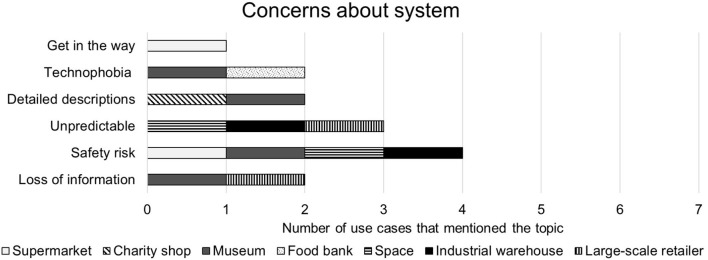
Summary of negative responses to the storage organization system.

Concerns expressed during interviews about the swarm system are given in Figure 8. The **Large-scale Retailer** was the only use case to state outright that it would not want this system. They said that their priority was stock control and they did not like that the individual agents would not be centrally controlled at all times. They also said that they thought that the swarm would require initial learning stages and they could not afford to have a system that was not good enough to work right away. This was not something that was given in the swarm system, but it is an opinion of swarm robotics that was felt before the interview. They also said that they did not like not knowing where the information and behavior was coming from at all times within the swarm. They stated that they felt a swarm would risk losing information that could create a disastrous fault within the warehouse management.

The most common concerning topic was safety with 4/7 interviewees citing it as a risk factor when working with robots. For example, the **Museum** said that battery fires and trip hazards were both safety risks in the proposed system. The **Space Industry** and **Industrial Warehouse** representatives were both concerned about the unpredictability of a swarming system as opposed to a directly controlled system. The **Museum** and the **Charity Shop** both said that they did not think that a robotic system of any kind would be able to give rich enough descriptions of stock to improve upon human workers. The charity shop did not think that the system would be able to price items because of this gap, but they were happy with the idea of a technology that worked alongside humans, where swarms would sort items and humans would check and price them. There were also worries expressed for the risk of loss of information due to technology failure (expressed by the **Museum**) and that volunteers or staff would not be able to work with the technology (expressed by the **Museum** and the **Food Bank**). The **Supermarket** employee said that they would like the system but were concerned that it would get in their way if it used drone technology. It should be noted that drone technology was in no way mentioned to the interviewee prior to this comment.

##### 4.2.4.4. Trust

The **Large-scale Retailer** said that they would not trust a swarming system because they would not be able to know the information about where everything was in the warehouse and why it was there at any time. This is compared to their current, centralized system which is heavily controlled. 6/7 of the use cases said that they would trust the system but most 4/6 had a condition to add to this statement. The **Food Bank** had no caveats and the **Supermarket** said that damage was already caused to their products so they thought that the system would only improve this rate of damage even if it made some mistakes and therefore they would trust it. The **Charity Shop** said that they would trust the system with sorting and handling items but they did not think it would be good enough to trust with pricing items without human supervision. The **Museum** was concerned about practical safety risks including the possibility of a collection piece being damaged if a robot collided with it. They said that if the system was proven to be safe then they would trust it.

The **Space Industry** said that it would trust the system if risk could be eliminated but the interviewees were split on how possible this would be. One representative said they considered swarms too unpredictable to ever be accepted in space applications where any mistakes can be mission critical. However the other representative said that they thought that swarms could be trustworthy if they were sufficiently developed and tested.

The **Industrial Warehouse** expressed that they were very interested in the system and would like to make it work but they would find it difficult to trust until it passed sufficient safety regulations. They expressed concern that this would be difficult, as no regulations currently exist.

### 4.3. Bridge Inspection Study

The focus groups conducted for the bridge inspection use case produced distinct themes as shown in Table 1. The details of these results are presented below.

**Table 1:** Distinct themes were identified in notes taken in the bridge monitoring focus groups.

**Task mentioned for robots**	**Positives**	**Concerns**
Collecting data to help plan human inspections	More data collected	Speed
Constructing 3D models of bridge	Possible time savings	Cost
Providing information on hard to reach areas	Cost of individual units	Safety of inspection
		Value of collected data Locomotion abilityRetrieval of units

*These were tasks participants thought robots could help with alongside positives and concerns participants had with the scenarios mentioned*.

#### 4.3.1. The Art of the Profession

The participants described the task of bridge inspection as finding and assessing defects in the structural components of a bridge. This assessment was said to be crucial to ensure the bridge was safe and could be maintained properly. This task is not trivial with expertise required for identifying, quantifying and determining the consequences of any defect. One participant mentioned a difficulty in finding people with such skills. Both groups operated in fairly distinct sectors with one primarily inspecting railway bridges while the other inspected a variety of short to medium sized road bridges. Multiple levels of inspection were mentioned. The first level was a general inspection in which the bridge areas which were easy to access were surveyed (mostly visually) every 2 years. The second level was a detailed physical inspection, known as a principle inspection, that was carried out every 6 years. The principal inspection required all bridge elements to be inspected at close range. These procedures are inline with industry standards outlined in Highways England (2017). The first group were primarily concerned with these types of inspections as their expertise were in special access measures. The second group administered both types of inspections on behalf of a local authority.

#### 4.3.2. Their Challenges

The challenge mentioned most by inspectors was accessing structural components of the bridge in difficult to reach areas. Participants described current measures such as rope-access and scaffolding as costly in terms of money and time. Participants of both groups also highlighted the diversity of the structures they have to inspect as another challenge. Each group described having to deal with bridges made from different materials and with different designs, many of which were built without any consideration of how they would be inspected.

Another challenge frequently mentioned by both groups was that inspections had to be carried out in a way which minimized the disruption to the traffic on the bridge. For the first group this was a consequence of the dangers involved in inspecting railway bridges such as passing trains and high voltage cables. This lead to small timeframes where inspections could take place. The second group highlighted that many of their bridges are essential links for rural communities and so closing the bridge would adversely effect these people.

#### 4.3.3. Positives About Bridge Swarm Systems

In the discussion of the scenarios, most participants were receptive to working with robots and using data gathered from robots to help inform inspections. One group in particular saw the value in using a swarm, similar to that in scenario 2, to do a thorough sweep across large structures that could help target the deployment of roped access teams by logging the positions of detected defects on an existing 3D model. Participants also viewed enclosed small spaces such as culverts as useful environments to deploy a swarm in. Participants mentioned how current robot inspection of these areas uses a CCTV camera attached to a caterpillar track chassis, but these are very expensive. Hence they liked the idea that a swarm system was more modular and so losing single robots would represent a small financial risk. However, they did not imagine the swarm would be able to inspect the culvert itself but could provide useful information before human teams enter. For example the swarm could provide a rough dimensional survey to detect collapsed sections, or sense if hazardous gases had accumulated.

#### 4.3.4. Concerns About Bridge Swarm Systems

The following are concerns expressed by the bridge inspection participants following a description of the scenarios. In this study, requirements for trust were not explicitly asked to participants, but the concerns expressed by participants indicate a lack of trust in some elements of the scenarios presented.

##### 4.3.4.1. Data value

The type of data gathered by any system was one concern raised frequently. Both groups viewed touching as an essential part of an inspection but not something they thought a robot would be able to emulate. They stated the importance of being able to sound parts of the structure with a hammer and examining the depth of any defect. Examples given included listening for hollow sounding areas which can indicate delamination in concrete or establishing the extent of paint flaking on steel elements. One participant also made the point that being able to identify issues in images of the structure came from doing this hands on work and this skill could be lost or diminished if the entire bridge inspection process was automated. While 3D models were viewed as a useful deliverable of a robotic system by participants, they also thought there were limitations with using them. They said that while potential defects could be identified using them, establishing their severity would often require visiting the defect in person as the model could not provide the same interactions as being on the structure in person. The groups also mentioned that changes in the condition of the structure were more important rather than one-off detections, such as in the second scenario. They thought robots would not be able to evaluate the severity of defects without knowing the previous state of the defect they had detected.

##### 4.3.4.2. Time and cost constraints

Both groups referred to time and cost constraints as a major factor in determining if a particular technology was valuable and whether they would use it. For example, a textured 3D model was viewed as useful, and if there was no cost it probably would be widely used. However, participants in the second group viewed the time and cost in obtaining such a model as too high for the number and size of the bridges they dealt with. In their opinion many areas on these types of structures can be documented in sufficient detail from the ground with a few photos, so deploying a robotic system to get a very detailed model is overkill. Hence technology was only viewed as valuable when the environment was more constricted or complex, since this cannot be obtained easily with current practices.

##### 4.3.4.3. Data processing

The amount of data that was collected and how it was processed was also highlighted. Both groups mentioned that a large amount of unprocessed data would not be helpful. For example, participants said that trawling through footage captured from robotic systems, or large collections of images had been tedious in the past. They also mentioned that structural health monitoring systems have this problem if not used precisely. This issue came up a lot in response to the second scenario, in which many robots covered the sides of the bridge detecting damage. Many participants stated that they imagined a system that captured data indiscriminately would flag up a large number of possible defects. Participants were then worried they would not have the resources to check each one during the inspection period. Some participants suggested the second scenario would be more useful if the system's output could be tied to a 3D model, as this would mean the data would not have to be checked in real time. In this case the system would simply collect more data about the structure than they do now. Both groups also highlighted that the top priority was identifying safety critical issues on the structure. Some participants felt a system that gathered more data, if processed properly, would help in this task. However, others felt that the robots performing damage detection would be challenging, a view supported by the general observations on structural health monitoring by Webb et al. (2015).

##### 4.3.4.4. Robot capabilities

Participants also had concerns about the abilities of the robots themselves, asking how they would move in such difficult environments. Many assumed the robots would need to fly and mentioned issues with using current inspection drones such as risks of collisions and flight restrictions. Another issue brought up was the retrieval of a large number of robotic units. Participants stressed that everything would need to be retrieved so that it would not contaminate natural habitats.

## 5. Discussion

Although the three studies featured different fields of application (fire and rescue, storage organization, bridge inspection), there were similar results and opinions across the participants. In this section, we highlight those similarities to help shape future responsible and successful deployments of robot swarms in the physical realm.

### 5.1. There Is Opportunity for Swarm Robotics in the Workplace

Participants across the three studies welcomed robots for certain tasks, especially robot swarms. For them, the main advantages are the ones related to robustness via redundancy (no single point of failure) and high performance due to the use of a large number of robots. In the case of the fire and rescue focus group, these properties would be helpful in scenarios that participants felt were most useful, as identified in the focus groups (real-time information gathering, dangerous tasks, communication channels, finding exit routes, testing for hazards/traps, victim location/tracking, tactical ventilation), and from the questionnaire (locating victims, risk/incident assessment, mapping the environment, communication links), as Driewer et al. (2005) also found in their study. In these applications, high speed and large area coverage are common aspects, hence benefiting from a robot swarm collectively performing them in parallel.

Similarly in the storage organization study, almost all of those interviewed said that their current sorting systems would benefit from additional autonomy and they welcomed robot swarms (with caveats and assurances). Many of the use cases said that having an automated sorting system using a swarm of robots would be desirable because it would allow them to perform other less tedious and more useful jobs at the shop front. Tasks that they projected the robot swarm could do, that were not part of their current capabilities, included taking automatic inventory which many interviewees stated would improve the efficiency of their warehouses. This extended in almost all cases (automated warehouse, food bank, supermarket, charity shop, museum, and space) to predictive ordering based on projected demand changes informed by customer patterns, weather forecasts etc.

The bridge inspection participants were receptive to any technology which could gain valuable information about the structure. However, the value of the information was crucial to the participants views of a swarm system. This value depended on the measurements being made, pictures could be used to identify defects but were less useful for characterizing their extent. The value also increased with the difficulty in accessing a given area by humans, such as confined spaces, or with increasing size of the structure, at which point human inspection becomes very slow and costly. The aspects the participants value also fit well with the swarm's abilities. For large structures, the area needing to be covered would be sizeable and suitable for a swarm's parallel operation. Enclosed spaces represent an unknown environment that would require the swarm's robust operation.

### 5.2. Identifying the “Art of Their Profession” Will Inform Tasks to Be Automated

There is a common theme across most participants in the three studies. They welcome technology that can assist them with certain tasks, but not all of them. In the fire and rescue study, participants' priority would be on robots that could assist them with information-gathering (e.g., locating casualties, mapping, communication links) or simple actions (clearing the way, lifting heavy things, tactical ventilation) with no autonomous decision-making process in place. This preference can be explained from two different points of view. On the one hand, participants pointed out that finding the fire/casualties and gathering information for their decision-making processes are the main challenges they face. On the other hand, they highlighted their fear that robots making autonomous decisions could cause more harm than good because of unforeseen consequences (many factors are in place during firefighting and rescuing), or lack of understanding of such decisions. Particularly interesting is their preference for not having fire extinguishing robots. Participants felt that there were many aspects involved in firefighting, and that only them, humans, would be capable enough to extinguish fires. This suggests there are certain aspects of their profession that they would not like automated, but done by humans—the art of their profession.

In the storage organization use case interviews, a concern from the workers was that swarm robots were not capable enough to sort the warehouse with full autonomy. For example, the Charity Shop workers said that they did not think that a robotic swarm system would be able to price the items correctly. They said that this is because when the human workers price items, it is a judgement that can be based on current trends, how the item feels, brands etc. In the same way, the Museum workers doubted that a robotic swarming system could replace human workers in being able to provide a detailed enough description of collection items to sort them. In this way, the art of the profession (i.e., the charity shop worker knowing from experience and instinct how much an item is worth) is something that workers consider to be an important part of the sorting process and not something they consider robots capable of doing without a human.

The bridge inspection study found that participants also doubted the robots' abilities to evaluate things, in this case the condition of structural members. They stated that touching and sounding the structure are essential for finding the extent of any defects identified. Additionally many defects needed to be evaluated over time to determine their severity, hence a robot which is only measuring some quantity at one time point would not be able to quantify its seriousness. Participants agreed they would rather have robots supporting their decision-making processes as much as possible, but not acting autonomously when it comes to making decisions. Takayama et al. (2008) also found that robots were not preferred for occupations that require evaluation and judgement. Semi-autonomy, meaning that robots can perform some tasks by themselves but always subject to human input (human in the loop), is the preferred mode of operation. Semi-autonomy was also the preferred mode of operation in the study done by Driewer et al. (2005).

Robots are often negatively portrayed as machines taking over jobs. The fact that there are some aspects of their profession that participants would like to protect could seem to be related to this, although a direct question about fear of losing their jobs was not asked to participants. Participants broadly welcomed the use of robots in their jobs, and agreed they would be a tool to enhance/assist in their operations rather than a replacement. This is similar to the findings of the survey by Takayama et al. (2008) in which non-expert participants were more likely to prefer robots in a given occupation with people, rather than instead of people. Taking into account that there are barely any robot swarms currently in place in the professions explored in this paper, the fact that participants welcomed their use for certain tasks shows a high degree of preliminary acceptance. Therefore, when looking at how to best deploy robots in the physical realm, it is important to identify with end users which aspects are/are not desired to be automated to increase user acceptance.

### 5.3. Tackle Concerns to Increase Acceptance and Trust

Participants were mainly positive about robot swarms and the applications in their fields. However, there were caveats in each case, meaning that participants would trust swarm robotics systems under certain conditions. It is then crucial to address these concerns, if a successful implementation in society is sought. In fact, user acceptance and trust have been identified as the major bottleneck when taking robots to real-world applications (Kruijff et al., 2014).

#### 5.3.1. Transparency and Accountability

In the study with fire and rescue services, participants pointed out that robot swarms should always store all the data they generate/process—timestamped. It is very important for them to understand *what* the swarm is doing, especially in case an investigation is required. In this sense, the swarm must be accountable, i.e. able to be queried and return a human-understandable answer. This is the concept of an ethical black box, described by Winfield and Jirotka (2017) as a mechanism to improve public trust by designing robots with accountability at the core.

Storage organization use cases were also concerned about the risk of loss of information and unpredictable behaviors. This was particularly concerning for the Large-scale Retailer and the Museum who both store millions of products in one place and can therefore not afford to lose control of their stock. Other use cases with fewer active stock pieces were more willing to experiment with new technologies because the risk of loss of information if the system were to go down is not as great.

In the bridge inspection study, transparency and accountability were not mentioned directly by the participants when talking about the swarm scenarios. This may have been due to the described data collection task not involving the swarm taking substantial decisions that would need explaining to bridge inspectors. Participants also expressed doubts over the individual robots' ability to make substantial decisions such as evaluating detected defects. Whether participants maintained this view in the case of the system being made fully accountable was not investigated.

#### 5.3.2. Reliability and Safety

For firefighters, another aspect to help build trust in robot swarms is reliability, i.e., the guarantee that if the robot swarm is deployed in a fire and rescue operation, it will work properly. In the scenarios they face, faults cost lives. Hence, all the information that the robot swarm might gather or the actions they perform must be completely accurate. This requires thorough verification and validation of the swarm robotics system before deployment. However, predicting the emergent collective behavior of robot swarms given the individual rules of each robot, and making sure that it is the only behavior that the swarm shows is a major challenge (Dixon et al., 2012). Further research on designing reliable swarms should be prioritized to increase trust, as well as reduce the number of risks arising from the use of swarms (Harbers et al., 2017).

Safety also came out as another requirement for acceptance and trust in the focus group with fire brigades. The robot swarm not becoming a physical obstacle (either for firefighters or casualties) was especially regarded as a crucial feature of the swarm robotics system. As argued above, this has to do with the requirement for robots not being detrimental to their operations. All in all, “technology is, in general, trusted if it brings benefits and is safe and well regulated” (Winfield and Jirotka, 2018).

The storage organization use case interviews found that safety was an important concern but of varying degrees. For example, the Museum cited worries about battery fires and trip hazards but it was not overly concerned about them since they are easily avoidable. On the other hand, the Space Industry representatives stated that missions are safety critical and therefore any technology that is included would have to have all risk removed before deployment could be achieved. They said that although they are interested in future developments of swarm robotics and its usefulness in space applications, they perceive its unreliability at this stage of development to be too high a safety risk to be viable for space missions. Both the Space Industry representatives and Industrial Warehouse stated they could not accept swarm technology until it passes safety regulations that are specific to swarm technologies.

Safety and reliability were also a primary concern of the bridge study's participants. For reliability, participants were concerned about how the swarm individuals would move over the structure or inside an enclosed space without getting stuck. They were also concerned about how the individuals could be retrieved given the lack of a tether. There were other concerns related to robots falling or hitting things such as people, high voltage cables or traffic. Although, it should be mentioned other bridge inspection technologies such as drones, scaffolding, and roped access are not without their own risks (Dorafshan and Maguire, 2018). These safety concerns indicate that for swarm technologies to be accepted in the future, relevant safety standards will need to be developed (Winfield et al., 2004; Bjerknes and Winfield, 2013; Beltrame et al., 2018).

#### 5.3.3. Ease of Training, Use, and Maintenance

Finally, most participants across the three studies agreed that they would trust the robot swarm assisting them at work as long as it was easy to learn about, use and maintain. In the study with firefighters, time is a crucial aspect for them. Hence, they require a system that can be deployed fairly quickly (ready by the time they arrive to the incident location), not too complex to use (their cognition abilities are harmed when firefighting, for example) and that does not require complex maintenance (always ready to be used). This places the focus on the scalability and adaptability of the robot swarm operations. Essentially, this means that if an action has to be done on the swarm, it should be independent of the number of robots in the swarm or the location of deployment.

Many of the storage organization workers interviewed said that their staff are volunteers and/or do not have a lot of spare time to train in how to use technologies. For this reason, out-of-the-box swarming systems would be needed to reduce set-up and maintenance during use. Any human-swarm interface would need to be very intuitive with little need for technology skills (for example, the museum said that their volunteers struggle with basic computer skills so they avoid technological solutions). In the bridge inspection study, participants were concerned with operating in tight cost and time constraints and so would also benefit from easy to use systems.

These results are in line with the findings from Yanco et al. (2006), where participants expressed their desire for the system to be easy to use—in fact, the system being difficult to use was the main cause for their test missions failing. Moreover, participants from the study led by Driewer et al. (2005) preferred an easy-to-use system. Authors then suggested having the ability to select different layers of information depending on what the specific user might require. This could indeed improve adaptability of the systems to users.

### 5.4. Mutual Shaping Can Facilitate the Deployment of Robot Swarms in the Physical Realm

The analysis of the responses to the pre-questionnaire and post-questionnaire in the study with fire brigades was used to understand the role of mutual shaping through focus group discussions in changing their opinions. The following changes in attitudes were noticed:

- **More tasks for robots:** In terms of tasks where robots could be useful, there was an overall increase in all tasks after the session (except extinguishing). This tells us that the session made them see how robots could be used for more tasks than they previously had thought.
- **Acceptance of robots increased:** There was over 20% increase to the *very likely* or *extremely likely* responses to the question related to acceptance of assistance from a robot. A total of 18 participants ticked either of these options in the pre-questionnaire, whereas 22 participants ticked participants ticked in the post-questionnaire.
- **A large swarm of robots is preferred**: When firefighters were asked about the number of robots they would rather have assisting them, *a few* was the most selected answer (13 participants), whereas *many* was chosen by only 3 participants. After the session, nine participants ticked *a few* and 10 participants ticked *many*.

Mutual shaping has been shown to be a successful way to engage in a two-way conversation with potential users and incorporate societal choices into the research and development process. If robot swarms are to be used in real-world applications, it is important to listen to all the parties who will be affected by it in the future. Almost three quarters of the firefighters said that they would like to be involved in the research and development process from the very beginning, in both questionnaires.

## 6. Conclusion

Robot swarms have been demonstrated performing a variety of tasks under laboratory conditions. However, potential users' exposure to the technology is limited. This has led to a number of unanswered questions around what people's perception of swarm robotics is, how comfortable people would be using the technology and what tasks they would like the technology to perform. In this work, three studies with a total of 37 potential swarm users were performed across three different sectors: fire and rescue, storage organization, and bridge inspection. Each study used participatory design style discussions that were structured to develop an understanding of each user's profession before introducing them to swarm robotics and discussing potential assistive swarm systems. It was found there was a generally positive reaction to robot swarms, but also some caveats. In both the fire and rescue and bridge inspection studies, participant's desired systems which would gather information to help inform human decisions. For the storage organization sector, a system which would sort stock and manage inventory in a space efficient manner was desired. Moreover, a common theme across the three studies was that there are some aspects of their jobs (especially when it comes to decision-making) that participants would not like to be done by autonomous robots. We call this *the art of the profession*. Therefore, it is important to identify with end users which aspects should be automated, and which should not, to increase users' acceptance. The caveats found were either due to doubts about the system's capabilities compared to a human or trust in its operation. To improve trust and acceptance in swarm systems in the future participants highlighted a number of areas including: transparency, accountability, safety, reliability, ease of maintenance and ease of use. Finally, it was shown that designing the study with personnel from fire and rescue services following a mutual shaping approach positively changed their opinions about robot swarms assisting them.

Because swarm robotics technology is still being developed, now is the perfect time for swarm robotics researchers to create a link with users to identify what needs to be done to build trust and to ensure the technology is fulfilling a desired role. This will facilitate the deployment of robot swarms in the physical realm.

## Data Availability Statement

The datasets generated for this study are available on request to the corresponding author.

## Ethics Statement

The studies involving human participants were reviewed and approved by Research and Enterprise Development (RED) at University of Bristol and Research Ethics at the University of West England. The participants provided their written informed consent to participate in this study. Written informed consent was obtained from the individual(s) for the publication of any potentially identifiable images or data included in this article.

## Author Contributions

DC-Z designed, recruited participants and facilitated study with personnel from fire and rescue services, analyzed transcripts and questionnaires, and substantially contributed to the writing of the manuscript. EM designed, recruited participants and facilitated study with personnel from storage organization, and substantially contributed to the writing of the manuscript. JH designed, recruited participants and facilitated study with personnel from the bridge inspection industry, and substantially contributed to the writing of the manuscript. GT assisted DC-Z during one focus group, and transcribed audio recordings. PV and SH made substantial contributions to the conception of the study with the bridge inspection industry, and assisted with participants recruitment. MG and SH made substantial contributions to the conception of the storage organization study, and assisted with participants recruitment. AW and SH made substantial contributions to the conception of the fire and rescue study, and assisted with participants recruitment. MS supported the storage organization study, including but not limited to the funding of the storage organization project via Toshiba Research Europe Limited. PV, MG, AW, and SH also critically revised the work.

## Conflict of Interest

MS was employed by the company Toshiba Research Europe Limited. The remaining authors declare that the research was conducted in the absence of any commercial or financial relationships that could be construed as a potential conflict of interest.
