# Supplementary material for: Mutual Shaping in Swarm Robotics: User Studies in Fire and Rescue, Storage Organization, and Bridge Inspection
Source: Front Robot AI. 2020 Apr 21;7:53. doi: 10.3389/frobt.2020.00053 (PMC7806009; doi:10.3389/frobt.2020.00053)
Supplement: Supplementary file 1 [file Data_Sheet_1.PDF]

Supplementary Material

1 METHODOLOGY - EXTENDED

1.1 Fire & rescue study

Focus groups with fire & rescue services were designed to have the following structure:

1. **Art of their profession.** The following questions were verbally asked to participants to understand their jobs and processes:
   a. What is your area of work?
   b. What are the typical tasks in your job?
   c. What tools do you currently use?
   d. What technologies have been introduced into firefighting and rescuing?
   e. What do you think about using robots as a tool to help in your job?
   f. In which firefighting/rescuing tasks would robots be most useful?
2. **Introduction to swarm robotics and possible scenarios of application.** Facilitator used presentation slides on a computer to present the following:
   a. **Current state of robots for firefighting/rescuing**
      An overview of recent approaches suggested by the research community was given to participants to show the current state of the art of robots for firefighting/rescuing, as well as their limitations (e.g. bulky robots that may become an obstacle).
   b. **International competitions for multiple robots**
      The researcher described the ERL Emergency Service Robots competition held in Italy in 2017 (Winfield et al., 2017), and the Swarm and Search AI 2019 Fire Hack held simultaneously in the UK and US in 2019. This was used to show international efforts in promoting research for robots assisting in fire and rescue operations.
   c. **Explanation of swarm robotics**
      A video explaining the main concepts of swarm robotics (a large number of robots performing a collective behaviour in a decentralised fashion without any leader, inspired by natural swarms) was shown to participants (SciShow, 2017).
   d. **Possible scenarios of application.** Four different scenarios were shown one by one using presentation slides on a computer.
      (1) **Indoor sensing**
         This scenario depicts a swarm of (aerial) robots exploring a building on fire and creating a heat map of the building plus the location of firefighters and hazards.
      (2) **Indoor communication paths and exit routes**
         A swarm of (aerial) robots is shown inside a building. In this case, the swarm has located a casualty in one part of the room and it is maintaining a communication link between the entrance of the building (representing the firefighters base station) and the casualty. In addition, robots in the shortest path between the entrance and the casualty are lighting up to show they are on the exit route.
      (3) **Indoor fire extinguishing**

In this scenario, a swarm of robots (ground, moving balls designed by one of the authors, and similar to Sphero^{TM} robots) is released inside a building on fire. The swarm explores the building, finds the fire and extinguishes it by releasing an extinguishing cargo.

(4) **Outdoor wild fire extinguishing**

In this final scenario, a drone releases a swarm of robots (ground, moving balls) near a wildfire in a forest. The swarm moves to the fire and extinguishes it like in the previous scenario.

3. **Discussion of scenarios of application.** Participants were verbally asked the following questions:
   a. Can you think of robot swarms being used in other scenarios/for other tasks?
   b. Would you rather use only one robot, a few robots or a very large swarm?
   c. Would you rather use completely autonomous, semi-autonomous or teleoperated robots?
   d. What are the challenges for robot swarms to become a tool in firefighting/rescuing?
   e. What requirements for such a robot swarm would you have?

In addition to the previous parts, a paper-based questionnaire was handed out to participants before the start of the session and at the end of it. The same questionnaire and format was used as pre- and post-questionnaire to quantitatively measure their attitudes towards robots in firefighting and rescuing before and after the focus group, respectively. They were given as much time as they needed to answer the following questions:

1. **In your opinion, how useful could robots as a firefighting/rescuing tool be in the future?** (single choice): Not at all useful, slightly useful, moderately useful, very useful, extremely useful.
2. **In which firefighting/rescuing tasks would robots be most useful?** (multiple choice): Risk/incident assessment, mapping the environment, clearing the way, extinguishing fire, locating victims, rescuing victims, communication links, other (please specify).
3. **How likely would you be to accept help from robots in your job?** (single choice): Not at all likely, slightly likely, moderately likely, very likely, extremely likely.
4. **In your opinion, how many robots would be most useful for firefighting/rescuing?** (single choice): None, only one, a few (no more than a few dozens at the most), many (hundreds, thousands or more).
5. **Helper robots for firefighting/rescuing would be most useful in what mode of operation?** (single choice): When fully controlled by human experts, when semi-autonomous (responsive to human experts' instructions), when fully autonomous performing a pre-defined task (for example, extinguishing fire).
6. **When do you think fire brigades should be included in the research and development process of helper robots for firefighting/rescuing?** (single choice): Not necessary ever (fire brigades only as consumers of the product in the market), from the testing stage (after a prototype has been developed), from the very beginning of research and development (the design stages).

1.2 Storage organisation study

The storage organisation study had the following structure:

1. **Art of their profession.** The following questions were designed to gather information about the current processes within the use case stock rooms and how they organise their stock. This includes the tasks that require specialist systems or skills, specific to that profession and how swarms might fit into this.

a. **What processes do you currently use to organise your stock?** This question aimed to discuss the lifecycle of stock through the sorting processes from when the items are delivered to when they are moved to the shop front or packaged up. Prompts encourage participants to contribute details such as: tools they use (software, equipment, etc.); inventory processes (how often, scanning tags, etc.); categories of stock; and feelings about the process such as difficulties encountered. It also includes enquiring for more information about how the worker feels about the process such as "What are the difficulties in the current processes you use?".

b. **What tasks would you like a robot to help you with?** This question was asked before any information was given about swarm robotics or scenarios of application. Prompts encouraged participants to think about aspects of their job they do not like.

2. **Introduction to swarm robotics and possible scenarios of application.** Information about what swarm robotics is and a brief description of the state of the art was given to participants. Then, a spoken description of an out-of-the-box swarming system was given to the participants in the context of their storage facilities and stock items. The following is a summary of the description of the system given to the participants:
   - The system is made up of a swarm of robots. The benefits of swarms include: no single point of failure; no changes needed to be made to the programming for varied conditions or items; scalability; intelligent adaptation of behaviour to changing demand.
   - The system is a black box so the user does not interact directly with the stock or the organisational system.
   - The stock is provided to the system which sorts it into an efficient storage system.
   - Items can be requested by the user and are presented by the system.
   - Information about the stock (such as inventory) is collected by the system and presented to the user.
3. **Discussion of scenarios of application.** The following questions were in the context of their personal work in their stock room and what parts of the proposed swarm system would be most and least useful to them.

   a. What do you think is most helpful about the black box swarm system?

   b. What do you think would be the disadvantages to the black box swarm system?

   c. Would you trust this black box swarm system?

   For 3(a), 3(b) and 3(c) additional questions were used to understand their attitude to both the swarm part of the system and the automatic sorting system itself. Both of these elements were important to the results and were asked in order to understand, for example, if the idea of a swarm changed the attitudes towards the system itself.

1.3 Bridge inspection study

This session was structured as follows:

1. **Art of their profession.** The participants were asked what their roles were in the inspection process and what challenges they faced in their role. They were also asked if they currently employed any robotic systems in their operations.
2. **Introduction to swarm robotics and possible scenarios of application.** A spoken description of swarm robotics was given by the researcher. This consisted of explaining how in nature large numbers

of individually limited creatures can perform a larger task by following local rules about how they interact. Then saying that these ideas can be applied to robotics by having a system composed of many simple robots that also interact and perform a task that humans desire to be done. Following this, an explanation of two swarm robotic systems was given. The participants were asked what their impression of each one was and their opinions were noted. The first scenario was described as follows. A hundred small robots are released into an enclosed area of the bridge such as a box girder. As they move they capture hundreds of images which can be used to recreate a 3D model of the area. This scenario produces a tangible deliverable, a textured 3D model. This is a similar output to previous robotic approaches (see Khaloo et al. (2018)). The second scenario was described as system where a thousand robots are released to crawl over the exterior parts of the bridge. When they detect damage they will stop and alert inspectors to the damage by changing the colour of an onboard light. These spots could later be checked by an inspector to evaluate the detected damage.This scenario provides the users with a system which aims to focus the attention of inspectors during an inspection by performing damage detection, which is arguably the most difficult structural health monitoring task but also the most valuable (Webb et al., 2015).

3. **Discussion of scenarios of application.** Each scenario was then discussed, with participants giving their views on if they thought a particular system would be useful and detailing its challenges.

REFERENCES

Khaloo, A., Lattanzi, D., Cunningham, K., Dell'Andrea, R., and Riley, M. (2018). Unmanned aerial vehicle inspection of the placer river trail bridge through image-based 3d modelling. *Structure and Infrastructure Engineering* 14, 124–136. doi:10.1080/15732479.2017.1330891

[Dataset] SciShow (2017). The coming robot swarms. Youtube video accessed at `https://www.youtube.com/watch?v=uAAYivTtOpQ` on 18/11/19

Webb, G. T., Vardanega, P. J., and Middleton, C. R. (2015). Categories of shm deployments: Technologies and capabilities. *Journal of Bridge Engineering* 20, 04014118. doi:10.1061/(ASCE)BE.1943-5592.0000735

Winfield, A. F., Franco, M. P., Brueggemann, B., Castro, A., Ferri, G., Ferreira, F., et al. (2017). eurathlon and erl emergency: A multi-domain multi-robot grand challenge for search and rescue robots. In *Iberian Robotics conference* (Springer), 263–271
